# How to deal with Substance Abuse in a Telepsychiatric Setting

**DOI:** 10.1192/j.eurpsy.2022.184

**Published:** 2022-09-01

**Authors:** G. Dom

**Affiliations:** University of Antwerp, Psychiatry, Boechout, Belgium

**Keywords:** alcohol, tele-psychiatry, substance abuse, digital

## Abstract

**Abstract Body:**

Alcohol and (illicit) substance abuse are among the most common psychiatric disorders within the general population and their impact can not be underestimated. Reputedly for these disorders, there is a large treatment gap and treatment delay, i.e. large numbers of afflicted individuals never receive appropriate treatment and if they do so often many years after the onset of the disorder. The Covid19 pandemic has only aggravated these gaps. In many countries, due to the Covid 19 pandemic and its associated restriction measures telepsychiatric tools have become increasingly implemented (and funded) as regular parts in the possibilities in delivering interventions. With respect to substance abuse treatment, a vast body of research already showed promises both in the field of telepsychiatry as broader the use of digitalization (e.g. the use of virtual reality designed treatment interventions, digital monitoring). In the current presentation, an overview will be presented of both telemental health interventions and digital tools/interventions in the field of substance abuse treatment.

**Disclosure:**

No significant relationships.

